# Triphenylphosphonium Modified Mesoporous Silica Nanoparticle for Enhanced Algicidal Efficacy of Cyclohexyl-(3,4-dichlorobenzyl) Amine

**DOI:** 10.3390/ijms231911901

**Published:** 2022-10-07

**Authors:** Ho-Joong Kim, Sung Tae Kim, Dae Beom Park, Hoon Cho, Md Asadujjaman, Jun-Pil Jee

**Affiliations:** 1 Department of Chemistry, Chosun University, Gwangju 61452, Korea; 2 Department of Pharmaceutical Engineering, Inje University, Gimhae 50834, Korea; 3 Department of Nanoscience and Engineering, Inje University, Gimhae 50834, Korea; 4 College of Pharmacy, Chosun University, Gwangju 61452, Korea; 5 Department of Polymer Science and Engineering, Chosun University, Gwangju 61452, Korea

**Keywords:** mesoporous, silica, nanoparticle, algicidal activity, algicidal agent, cyclohexyl-(3,4-dichlorobenzyl) amine, triphenylphosphonium, algae, harmful algal blooms

## Abstract

Mesoporous silica nanoparticles (MSNPs) have been widely used for the delivery of different hydrophilic and hydrophobic drugs owing to their large surface area and ease of chemical alteration. On the other hand, triphenylphosphonium cation (TPP+) with high lipophilicity has a great mitochondrial homing property that stimulates the internalization of drugs into cells. Therefore, we designed a TPP-modified MSNP to enhance the algicidal activity of our new algicidal agent cyclohexyl-(3,4-dichlorobenzyl) amine (DP92). In this study, algicidal activity was evaluated by assessing the growth rate inhibition of two harmful algal blooms (HABs), *Heterosigma akashiwo* and *Heterocapsa circularisquama*, after treatment with DP92-loaded MSNP or TPP-MSNP and DP92 in DMSO (as control). For *H. akashiwo*, the IC_50_ values of TPP-MSNP and MSNP are 0.03 ± 0.01 and 0.16 ± 0.03 µM, respectively, whereas the value of the control is 0.27 ± 0.02 µM. For *H. circularisquama*, the IC_50_ values of TPP-MSNP and MSNP are 0.10 ± 0.02 and 0.29 ± 0.02 µM, respectively, whereas the value of the control is 1.90 ± 0.09 µM. Results have indicated that TPP-MSNP efficiently enhanced the algicidal activity of DP92, signifying the prospect of using DP92-loaded TPP-MSNP as an algicidal agent for the superior management of HABs.

## 1. Introduction

Harmful algal blooms (HABs) cause critical problems in the marine environment and fishery industry [1]. To manage HABs, several methods such as chemical algicides, clay flocculation, and usage of viruses or natural enemies have been used [2,3]. Chemical algicides are the most widely used methods. However, the use of chemical algicides in marine environments has limitations such as toxicity not only to HABs but also to marine life. One of the most common techniques for reducing HABs is the use of chemical compounds; however, their use was constrained due to their non-selectivity for other marine organisms and adverse effects on the ecosystem [2]. In a previous report, we developed substances that are both environmentally safe and specifically effective against HABs [4]. One substance of a dichlorobenzylamine derivative, cyclohexyl-(3,4-dichlorobenzyl) amine (DP92), presented a high algicidal effect against red tide with environmentally safe properties [4,5]. However, DP92 has a very high hydrophobicity and it is difficult to directly use DP92 in an aquatic environment. Therefore, the solubilization of DP92 is needed to enhance its algicidal efficacy [4]. We reported the solubilization of DP92 by cationic liposome. Not only the cationic liposome but also emulsion and polymeric micelle showed improved solubilization and enhanced algicidal activity. However, previously studied formulations are difficult to commercialize because the phospholipids used in the formulations are very expensive and unstable during storage [4]. Lower cost, ease of chemical functionalization and manufacturing, and dry storage ensured stability were primary requirements for the next delivery system for DP92. Mesoporous silica nanoparticles (MSNPs) were considered as a candidate.

MSNP has been considerably explored and used in the field of nanomedicine and biotechnology [1,6]. MSNP is characterized as hundreds of empty channels (mesopores) containing solid materials that are organized in a 2D arrangement of honeycomb-like porous structures resulting in a very large surface area (up to 900 m^2^/g) [7]. It can load large quantities of drugs owing to the large surface area, large pore volume, and adjustable pore size [1,8]. The property induces research into the delivery of various materials, including drugs, peptides, proteins, and nucleic acids [9,10,11,12,13,14]. It was revealed that these particles have internal and external surfaces, and the functionalization of these surfaces can be performed selectively and elegantly with numerous inorganic and organic groups [15]. MSNP has numerous benefits: it is inexpensive, easy to manufacture, and modifies surfaces chemically. More importantly, it is possible to use MSNP for hydrophobic drug delivery because of its hydrophobic pores. Additionally, surface-engineered MSNPs can be efficiently internalized by plant and animal cells without any cytotoxic effects in vitro [16]. Since the cost of using MSNP is lower and preparation is easier than that of phospholipids in other formulations (emulsions, liposomes, and polymeric micelles) and has a high drug-loading capacity, we selected MSNP as a new delivery system for DP92. 

A unique biological field known as “mitochondrion medicine” has developed recently, focusing on the mitochondrion as a crucial pharmaceutical target in the treatment of numerous serious diseases such as cancer [17]. This has to do with the growth of the understanding of mitochondria’s physiological functions. They are thought to mediate a number of significant biological processes via generating energy and managing the programmed cell death. Notably, mitochondria play a role in the biology of cancer through many mechanisms that promote cell proliferation [18]. Consequently, therapy that targets the mitochondria using nanoparticles might be a novel approach to resolving this issue. This situation led to the development of mitochondria-targeted MSNs through a number of tactics. The functionalization of carriers with lipophilic triphenylphosphonium (TPP) cations, which exhibit cell selectivity and penetration capacity due to poor solvation of the bulky head group, is the most efficient technique to produce mitochondria-targeted carriers [19]. The selective action of medications on the mitochondria of cancer cells is achieved using this technique. This is based on the well-known fact that the TPP cation concentrates the carriers on tumor cells, which have higher permeability than normal cell lines [20]. Notably, a delocalized charge on a lipophilic phosphonium cation shows strong attraction for negatively charged membranes. This property gives TPP-modified carriers efficient cellular absorption and mitochondria-targeting abilities. TPP is a lipophilic cation due to its three phenyl groups. Since TPP has sufficient cationic charge and hydrophobicity, it can easily attach and pass through the cell membrane, which is composed of a phospholipid bilayer, and it is possible to migrate into the mitochondria due to charge interactions between the positively charged TPP and the negatively charged mitochondria. Mitochondrial targeting is enhanced with a TPP group modification of small molecules. Therefore, TPP can be used to promote the internalization of materials into the cell [21,22]. 

We modified the MSNP surface with TPP. TPP-MSNP can effectively stimulate the delivery of DP92 into HABs. Two features of TPP-MSNP make it possible to deliver DP92 into HABs. DP92 can be applied to aquatic environments owing to the solubilization of MSNP. MSNPs modified with TPP can target HABs and permeate algal cell walls owing to the hydrophobicity and positive charge characteristic of TPP [23]. Thus, we have designed a new formulation where DP92 is entrapped by TPP-MSNP. 

In this study, the properties of MSNP and TPP-MSNP, such as particle morphology, particle diameter, polydispersity index (PI), zeta potential, and encapsulation efficiency, were evaluated. For the evaluation of TPP-MSNP internalization into algae, MSNP and TPP-MSNP labeled with coumarine-6 were used. Experiments of fluorescence absorbance analysis in algae, fluorescence microscopy observations around algae, and algicidal activity of DP92 in TPP-MSNP and MSNP were performed on *H. akashiwo* and *H. circularisquama*.

## 2. Results

### 2.1. Characterization of MSNP and TPP-MSNP

#### 2.1.1. N_2_ Sorption Analysis for Surface Area Change

The pore characteristics of MSNP-TPP were analyzed by measuring the nitrogen adsorption–desorption isotherm. The specific surface area of MSN-TPP was 264.88 m^2^/g, which is decreased by almost half of that (552.04 m^2^/g) of MSNP.

#### 2.1.2. Particle Properties

MSNP and TPP-MSNP were prepared according to a previously reported procedure [4]. For TPP-MSNP preparation, the amount of TPP per MSNP (100 mg) was estimated to be about 4.5 mg. MSNP and TPP-MSNP were fabricated with the expected particle diameter. As described in Table 1, the mean diameters of MSNP or TPP-MSNP were 126.43 ± 9.46 nm and 104.27 ± 2.05 nm, respectively. It was estimated that the particle diameter of TPP-MSNP was rather small; however, it was not significantly different, considering the standard deviations. The PI values of MSNP and TPP-MSNP were from 0.20 to 0.25, which suggests that both particles were fabricated with high homogeneity. It is known that the abundant Si–OH groups on the outer surface of MSNP cause the highly negative surface charges of MSNP around −40 mV [24]. MSNP and MSNP/DP92 showed negative charges of −31.67 ± 1.14 mV and −29.20 ± 0.95 mV, respectively. However, the cationic TPP modified MSNP and MSNP/DP92 presented a positive charge value of 25.41 ± 1.53 mV and 26.63 ± 1.07 mV, respectively. Dynamic light scattering (DLS) and zeta potential measurements showed slight changes in particle diameter and zeta potential owing to the presence and absence of TPP; however, the difference was not significant (Table 1).

In order to investigate the optimal feeding amount of DP92, the amount of feeding drug was changed, and the amount that showed the highest EE while feeding a small amount of DP92 was obtained. DP92-loaded MSNP or TPP-MSNP were prepared at various feeding amounts of DP92 (10, 15, and 20 mg) and their EEs were calculated as described in Section 4.7.4 “Determination of encapsulation efficiency of DP92-loaded MSNP”. As listed in Table 2, DP92 in MSNP and TPP-MSNP displayed a high EE of over 70%. The encapsulated amount of DP92 increased by increasing the feeding amount of DP92; however, when the feeding amount exceeded 15 mg, the encapsulation started decreasing. Considering the EE and encapsulation amount of DP92, the optimum feeding amount of DP92 was fixed at 15 mg for further study. 

**Figure 1:**
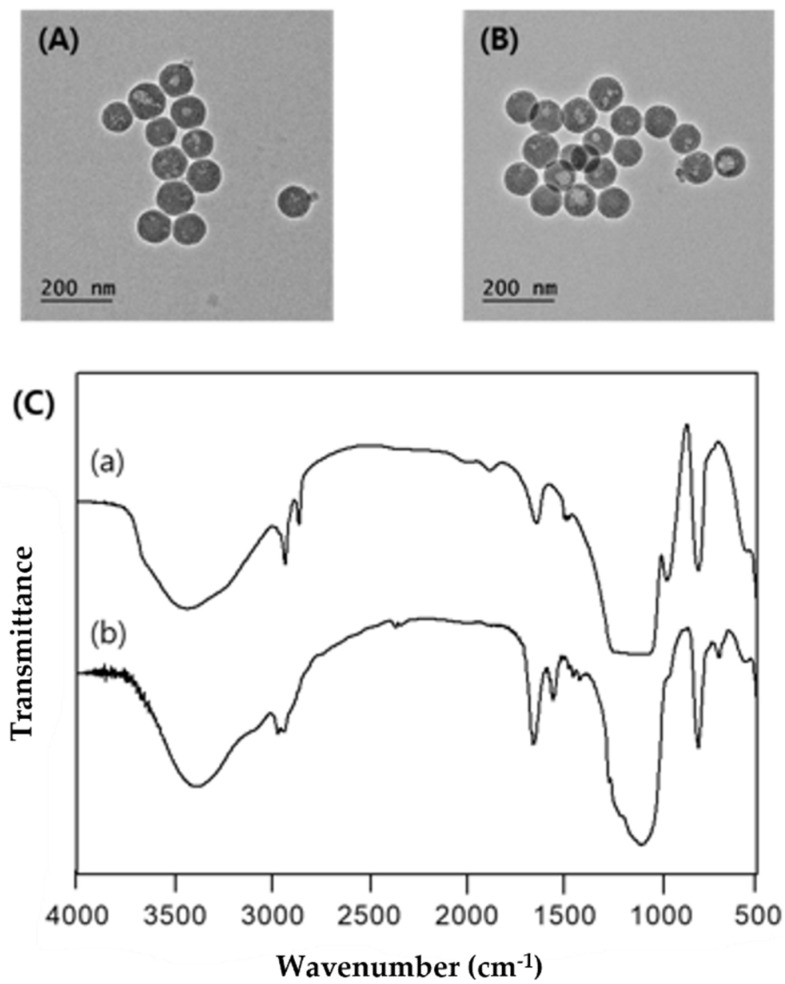
TEM image of (**A**) MSNP/DP92, (**B**) TPP-MSNP/DP92. (**C**) FT-IR spectra of (a) MSNP and (b) of MSNP-APTES-TPP.

Transmission electron microscopy (TEM) images were taken to identify the morphology and particle diameter of MSNP and TPP-MSNP. Figure 1A,B shows the morphologies of MSNP and TPP-MSNP, respectively. In Figure 1, the shapes of both MSNP and TPP-MSNP were similarly spherical, and their mean diameters were approximately 100 nm. 

The functionalization of silica samples is confirmed by FT-IR spectroscopy. The FT-IR spectrum of MSN-APTES-TPP, according to Figure 1C (b), presents the significant absorptions at 1455 cm^−1^ and 1415 cm^−1^, which are attributed to the stretching vibration of C=C in the monosubstituted benzene ring (TTP). The 960 cm^−1^ bands on the FTIR spectra disappeared, indicating that the MSN surfaces had been sufficiently aminated. The absorption band at 695 cm^−1^ is the deformation vibration ω of ring.

**Figure 2:**
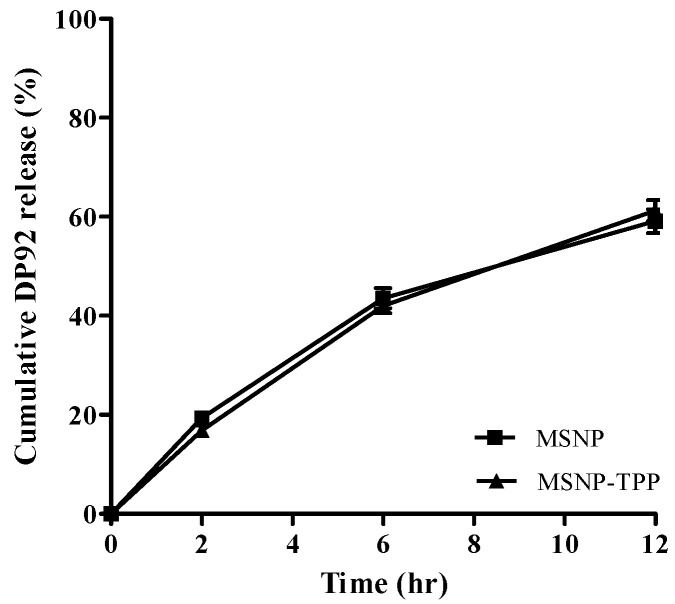
In vitro release profiles of DP92 from MSNP or TPP-MSNP. Data are presented as mean ± SD (*n* = 3).

The in vitro release results of DP92 from MSNP and TPP-MSNP are presented in Figure 2. When the systems were exposed to the media, 59.12% or 61.25% of loaded DP92 in MSNP or TPP-MSNP was released for 12 h without initial burst, respectively. 

### 2.2. Algae Internalization Property of MSNP and TPP-MSNP

**Figure 3:**
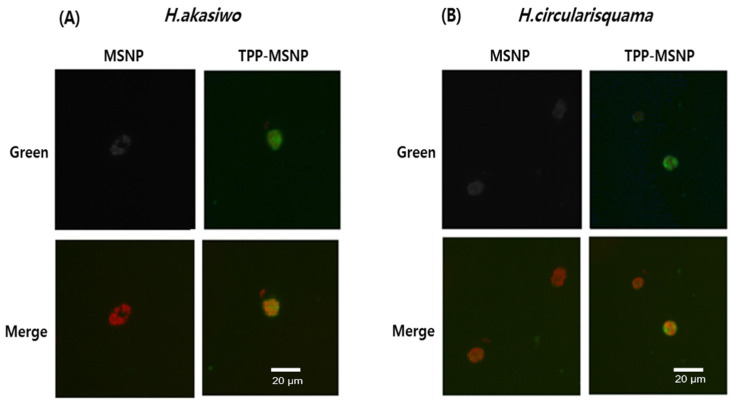
Fluorescence images of coumarin-6 labeled MSNP or TPP-MSNP on (**A**) *H. akashiwo,* (**B**) *H. circularisquama*.

In order to investigate the algae internalization property of MSNPs, fluorescence microscopy was performed. Two species of HABs (*H. akashiwo* and *H. circularisquama*) were employed as HABs models to identify algae-internalization activity. TPP-MSNP and MSNP (as control) labeled with coumarin-6 were incubated with *H. akashiwo* and *H. circularisquama* under algae culture conditions for 24 h. In the TPP-MSNP group, several green fluorescence spots were observed around and inside both HABs (Figure 3). As green fluorescence represented the coumarin-6 labeled TPP-MSNP, TPP-MSNP definitely existed around and inside the *H. akashiwo* and *H. circularisquama*. 

Fluorescence absorbance measurements were performed to quantitatively measure the degree of transition into algae. Like fluorescence microscopy, TPP-MSNP and MSNP were labeled with coumarin-6 and treated with *H. akashiwo* and *H. circularisquama*, and then incubated in algae culture conditions for 24 h. In addition, the amount of coumarin-6 that was isolated from the algae was measured for *H. akashiwo* and *H. circularisquama*. The fluorescence absorbance values of MSNP and TPP-MSNP for *H. akashiwo* were 120.49 and 171.46, respectively (Table 3). The values of MSNP and TPP-MSNP for *H. circularisquama* were 177.69 and 230.64, respectively (Table 3). 

### 2.3. Algicidal Activity of DP92-Loaded MSNP or TPP-MSNP

**Figure 4:**
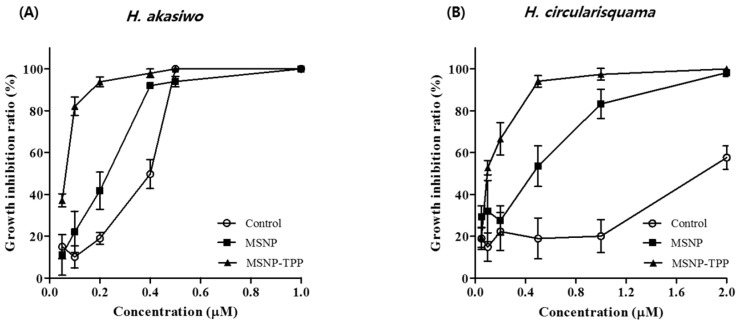
Algicidal activity of DP92 in MSNP and TPP-MSNP against (**A**) *H. akashiwo,* (**B**) *H. circularisquama.* Data are presented as mean ± SD (*n* = 3).

To perform the algicidal activity experiment, *H. akashiwo* and *H. circularisquama* were treated with DP92-loaded TPP-MSNP or MSNP and with DP92 in DMSO (as control) and incubated under algae culture conditions. After 24 h of incubation, live algae were counted. For *H. akashiwo*, the IC_50_ values of TPP-MSNP and MSNP were 0.04 ± 0.01 μM and 0.16 ± 0.03 μM, respectively, and that of the control was 0.27 ± 0.02 μM (Figure 4A and Table 4). For *H. circularisquama*, the IC_50_ values of TPP-MSNP and MSNP were 0.10 ± 0.02 μM and 0.29 ± 0.02 μM, respectively, and that of the control was 1.90 ± 0.09 μM (Figure 4B and Table 4). 

Furthermore, the IC_50_ value of TPP-MSNP was three times higher than that of MSNP for *H. circularisquama*. However, the IC_50_ of TPP-MSNP was four times higher than that of MSNP for *H. akashiwo*. The enhancement of algicidal activity with TPP was greater in *H. akashiwo*. The results suggest that *H. circularisquama* sensitively interacted with TPP, compared with *H. akashiwo*.

### 2.4. DP92 Internalization Efficiency of DP92-Loaded MSNP or TPP-MSNP

To identify that the enhanced algicidal activity in the TPP-MSNP group was due to the increase of DP92 delivery into the algae, DP92 internalization efficiencies were measured. For *H. akashiwo*, the internalization efficiency of TPP-MSNP and MSNP were 83.28 ± 6.00% and 53.00 ± 6.01 %, respectively (Table 5). The efficiency in TPP-MSNP was 1.6 times higher than that in MSNP. Similarly, for *H. circularisquama*, internalization of TPP-MSNP and MSNP were 50.94 ± 6.81% and 36.12 ± 4.24%, respectively (Table 5). 

As TPP is a material that can internalize into the mitochondria of living cells, it is possible to promote the transition into living cells. In fact, we have confirmed that DP92-loaded TPP-MSNP was transferred into the algae cell in the DP92 internalization efficiency experiments. This experiment showed that the enhanced algicidal activity of DP92 with TPP-MSNP was due to the increase of transition into algae with TPP-MSNP. However, this experiment did not show that DP92-loaded TPP-MSNP transits into the mitochondria of algal cells [25]. Therefore, further studies are required to identify the exact mechanism of the TPP effect.

## 3. Discussion

### 3.1. Characterization of MSNP and TPP-MSNP

Our fabricated MSNP had a negative surface charge of approximately −40 mV (Table 1), similar to the one reported earlier [24]. TPP is a lipophilic and cationic material, owing to its three phenyl groups. TPP was attached to the surface of MSNP by 3-aminopropyl triethoxysilane placed between TPP and MSNP. If TPP was well modified on the MSNP, TPP changed the anionic surface of MSNP to weakly negative or partially positive values [21,22,26,27]. Therefore, we anticipated that the surface charge of TPP-MSNP was at least neutral. As listed in Table 1, TPP-MSNP showed a positively charged surface, and the high value means that TPP was well attached on the anionic surface of MSNP and TPP-MSNP could interact with the algae cell surface that was negatively charged. 

The surface area of MSNP was related to the capacity for encapsulation of the drug. In particular, hydrophobic drugs can be physically adsorbed into pores placed on MSNP by van der Waals interactions and hydrogen bonding [23,24,28]. DP92 is a hydrophobic molecule and it is rarely soluble at 39.84 ± 0.95 μg/mL solubility in water [29]. Therefore, DP92 is a good candidate for encapsulation into hydrophobic pores on the surface of MSNP, and DP92 can be encapsulated at high concentrations into MSNP and TPP-MSNP. Despite having a surface area approximately half that of MSNP, TPP-MSNP encapsulated a comparable amount of DP92. This means that the surface area of TPP-MSNP and that of MSNP are large enough to encapsulate the initial amount of DP92.

The similar shape of MSNP and TPP-MSNP suggests that the modification of TPP onto MSNP did not affect the shape and diameter of the plain MSNP. Furthermore, the amount of DP92 released from the systems at each predetermined time did not differ significantly between MSNP and TPP-MSNP. This finding implies that the chemical modification of TPP had no effect on the release properties of DP92 encapsulated in MSNP. Therefore, the characterization data of MSNP and TPP-MSNP suggest that the TPP modification on MSNP did not affect the properties of MSNP and TPP-MSNP, except for the surface charge.

### 3.2. Algae Internalization Property of MSNP and TPP-MSNP

The results of the fluorescence absorbance measurement suggest that TPP-MSNP was successfully internalized into HABs. In contrast to the strong green fluorescence in the TPP-MSNP group, weak green fluorescence appeared around and inside the algae in the MSNP group (Figure 2). The spot around the HABs indicates the presence of TPP-MSNP containing coumarin on the surface of the HABs because the green spot suggests TPP-MSNP. Cationic TPP and the observed green spots of coumarin-labeled TPP-MSNP on the HAB surface suggest that the positively charged TPP-MSNP strongly interacted electrostatically with the HAB surface, indicating that MSNP does not interact with HABs; thus, the green spot was not found around the HABs. Regarding the charge, the specific difference between the TPP-MSNP and the MSNP makes this happen. With the cationic TPP, TPP-MSNP strongly interacted with the surface of HABs, which consequently enhanced the algicidal activity of DP92 [23]. The potential of the cationic TPP moiety to penetrate biological membranes is significantly aided by the high ionic radius it has as a result of resonance-mediated delocalization of the cationic charge on the phosphorus atom [30].

The results of the quantitative analysis of coumarin internalized into HABs also follow the aforementioned trend. In the *H. akashiwo* group, TPP-MSNP delivered 42% more coumarin compared with MSNP without TPP, whereas in the *H. circularisquama* group, TPP-MSNP delivered 30% more coumarin compared with MSNP without TPP. Therefore, it was found that, in both MSNP and TPP-MSNP, the fluorescence absorbance value for *H. circularisquama* was higher than that for *H. akashiwo*. This result suggests that *H. circularisquama* is more sensitive to foreign materials than *H. akashiwo*.

The green fluorescence of coumarin-6 labeled TPP-MSNP around and inside HABs and the higher fluorescence absorbance of coumarin-6 labeled TPP-MSNP suggest that TPP-MSNP was efficiently transferred by electrostatic attraction and cell-targeting effects. Therefore, the transition of DP92 with TPP-MSNP into HABs is also expected to be good, and the algicidal activity of DP92 with TPP-MSNP is expected to be improved.

### 3.3. Algicidal Activity of DP92-Loaded MSNP or TPP-MSNP

The reason for the high IC_50_ value in the control was due to the difficulty of transition into the algae owing to insufficient solubilization. However, in the MSNP and TPP-MSNP groups, sufficient solubilization occurred and DP92 was well transferred into algae. These results suggest that TPP-MSNP effectively targeted algae and enhanced the algicidal activity compared with MSNP. 

Like *H. akashiwo*, *H. circularisquama* also had lower algicidal activity in the control owing to insufficient solubilization. Similar to *H. akashiwo*, we also identified the effect of TPP on algae targeting and the enhanced algicidal activity for *H. circularisquama.*

The enhancement of algicidal activity with TPP was greater in *H. akashiwo*, indicating that nanoparticle uptake kinetics are cell dependent; nanoparticles have responded differently to different cells [31,32]. However, the mechanism is still unclear, and the results suggest that *H. circularisquama* sensitively interacts with TPP compared with *H. akashiwo*.

Owing to the solubilization of DP92 by MSNP, the algicidal activity of DP92 was enhanced up to 1.6-fold for both HABs compared with that of the control. Furthermore, the electrostatic interaction between the positively charged TPP-MSNP and the cell wall of HABs and the stimulation of penetration by TPP-MSNP might further enhance delivery of DP92 compared with the TPP-unmodified MSNP group, despite the rapid algae movement in the aqueous environment [33]. In fact, the TPP-MSNP group showed about three times higher algicidal activity than the MSNP group in both HABs. These results suggest that TPP-MSNP maximized the algicidal activity of DP92, owing to TPP. The advantages of TPP include the stability of the TPP moiety in biological systems, the combination of lipophilic and hydrophilic properties, the relative ease of synthesis and purification, the low chemical reactivity to cellular components, and the lack of light absorption or fluorescence in the spectral area of visible or near infrared (NIR) [34]. Moreover, TPP-MSNP is cost-effective compared with other substances (cationic phospholipids) used in previously studied formulation (emulsions, liposomes, and polymeric micelles) for solubilization [4]. Therefore, DP92-loaded TPP-MSNP is a suitable formulation for managing HABs as it is possible to commercialize and mass use DP92-loaded TPP-MSNP with an enhanced algicidal effect [35].

### 3.4. DP92 Internalization Efficiency of DP92-Loaded MSNP or TPP-MSNP

The DP92 internalization of TPP-MSNP was about 1.5 times higher than that of MSNP in *H. akashiwo*, whereas in *H. circularisquama* it was about 1.4 times higher than that of MSNP.

Furthermore, while MSNP and TPP-MSNP had similar properties for loading and releasing DP92, TPP-MSNP could deliver more DP92 to the inside of the algae due to the presence of TPP. The results suggest that the enhanced algicidal activity of DP92 with TPP-MSNP is due to the increase of the internalization into algae with TPP-MSNP. TPP-MSNP is a clearly effective tool for the delivery of DP92 into HABs, especially the positive charge, and the stimulated migration of DP92, owing to TPP, was very effective in enhancing DP92 algicidal activity. We can assume that the delivery of DP92 using nanoparticles such as MSNP can improve algicidal activity by solubilization. The results suggest that the enhanced algicidal activity of DP92 with TPP-MSNP is due to the increase of transition into algae with TPP-MSNP.

As TPP is a material that can internalize into the mitochondria of living cells, it is possible to promote transition into living cells. In fact, we have confirmed from the cellular internalization experiments that DP92-loaded TPP-MSNP was transferred into the algae cell. TPP cations are able to pass through the biological membranes of eukaryotic cells with ease due to their huge hydrophobic surface area and delocalized charge distribution, which reduces the solvation enthalpy [36]. The experiment showed that the enhanced algicidal activity of DP92 with TPP-MSNP was due to the increase of internalization into algae with TPP-MSNP. However, this experiment did not show that DP92-loaded TPP-MSNP transits into the mitochondria of algal cells [25]. Furthermore, normal cells’ mitochondria were shown to be difficult to stain. Because mitochondrial labeling is dependent on a highly negatively charged membrane potential, this finding implies that normal cells’ mitochondria were less active than malignant cells’ mitochondria [8,37]. Thus, it is not possible to predict whether TPP has targeted the mitochondria of algae cells or not. Therefore, further studies are required to identify the exact mechanism of the TPP effect.

## 4. Materials and Methods

### 4.1. Materials

Tetraethyl orthosilicate (TEOS), n-cetyltrimethylammonium bromide (CTAB), 3-aminopropyl triethoxysilane (APTES), 3-bromopropionic acid, triethanolamine, N-(3-Dimethylaminopropyl)-N-ethylcarbodiimide hydrochloride (EDC-HCl), N-hydroxysuccinimide (NHS), anhydrous dimethylformamide (DMF), toluene, methanol, acetonitrile, ammonium acetate, 3, 4-dichlorobenzaldehyde, cyclohexylamine, sodium borohydride, methylene chloride, and coumarin-6 were purchased from Sigma-Aldrich (St. Louis, MO, USA). All chemicals were used without further purification. 

### 4.2. Synthesis of DP92

Cyclohexyl-(3,4-dichlorobenzyl) amine (DP92) was synthesized as described in our previous report [4]. Briefly, 3, 4-dichlorobenzaldehyde (0.5 mg) and cyclohexylamine (282 mg) were mixed with methanol by stirring for 1 h at room temperature (30 mL). Sodium borohydride (161 mg) was added to the mixed solution. The mixture was then stirred at 25 °C until the starting materials that were identified using thin-layer chromatography (TLC) disappeared. The mixture was then extracted with methylene chloride and washed with water. The organic layer was dried over anhydrous magnesium sulfate and evaporated to obtain cyclohexyl-(3,4-dichlorobenzyl) amine (DP92). ^1^H NMR (300 MHz, CDCl3): δ 7.44 (d, J = 1.8 Hz, 1H), δ 7.38 (d, J = 8.0 Hz, 1H), δ 7.17 (dd, J = 8.0 and 1.8 Hz, 1H), δ 3.76 (s, 2H), δ 2.47 (m, 1H), and δ 1.91 (m, 10H).

### 4.3. Synthesis of TPP-(CH_2_)_2_-COOH

TPP–(CH_2_)_2_–COOH was prepared according to a modified procedure [38]. 3-Bromopropionic acid (0.765 g, 5.00 mmol) and triphenylphosphine (1.31 g, 5.00 mmol) were dissolved in anhydrous toluene (20 mL). The resulting mixture was heated to reflux under an argon atmosphere for 24 h and then cooled to ambient temperature. The mixture was concentrated under reduced pressure. The resulting precipitate was washed with ethyl acetate to produce a white solid (0.860 g, 41.4% yield). ^1^H-NMR (250 MHz CDCl_3_): δ 7.88–7.66 (m, 15H), 3.82–3.68 (m, 2H), and 3.15–3.01 (m, 2H) ppm. ^13^C-NMR (250 MHz, CDCl_3_): δ 172.3, 135.5, 1338, 130.7, 118.0, 116.8, and 28.23.

### 4.4. Synthesis of MSNP

The synthesis of MSNP was performed following a previously reported procedure [29]. A mixture of 1.53 g of cetyl-trimethylammonium bromide, 0.300 g of triethanolamine, and 100 mL of deionized water was stirred at 80 °C for 1 h, and then 14.45 g of tetraethyl-orthosilicate (TEOS) was added into the mixture. The mixture was stirred at 80 °C for another 2 h and then cooled to ambient temperature. After ultra-centrifugation, the precipitated MSNs were washed with ethanol and then stirred in HCl (36%)/methanol (5:45 v/v, 50 mL) for 24 h. After high-rate centrifugation, the precipitate was washed with ethanol and dried in an oven at 100 °C for 20 h to yield 960 mg of MSNP.

### 4.5. Synthesis of TPP-MSNP

The synthesis of TPP-MSNP was carried out following a modified method [39]. A total of 0.7 mL of 3-aminopropyl triethoxysilane (APTES) and 100 mg of MSNP were dissolved in 30 mL of ethanol and refluxed for 24 h. The particles were separated via centrifugation (10,000 rpm, 20 min), washed with ethanol, and dried in air at ambient temperature for one day. After the MSNP-APTES was dispersed in anhydrous DMF (10 mL), 40 mg of TPP–(CH_2_)_2_–COOH, 28.5 mg of EDC-HCl, and 17 mg of NHS were added to the mixture. The mixture was stirred at room temperature for 24 h. After high-rate centrifugation, the precipitate was washed with ethanol and dried in an oven at 100 °C for 20 h to yield 65.5 mg of TPP-APTES-MSNP (TPP-MSNP). To quantify the amount of TPP attached to the MSNP, UV/Vis absorption measurements using a standard calibration curve based on TPP–(CH_2_)_2_–COOH were taken. The supernatant in the washing process of TPP-MSNP synthesis was collected. The amount of unreacted TPP–(CH_2_)_2_–COOH in the supernatant was measured employing Beer’s Law regression of the standard calibration curve at 268 nm. The mass of TPP attached to MSNP was calculated by subtracting the mass of unreacted TPP–(CH_2_)_2_–COOH in the supernatant from the total mass of TPP–(CH_2_)_2_–COOH used for the synthesis step of TPP-MSNP.

### 4.6. Preparation of DP92-Loaded MSNP and TPP-MSNP

DP92 was loaded onto MSNP or TPP-MSNP using the modified preparation method [28]. Briefly, MSNP (5 mg) or TPP-MSNP (5 mg) was mixed with varying amounts of DP92 in 1 mL of methanol. The mixture was sonicated using a bath-type sonicator (Power sonic 420, Hwasin tech, Daegu, Korea) for 4 h, followed by centrifugation at 5000 rpm for 5 min. The supernatant was collected to analyze the concentration of DP92-loaded MSNP or TPP-MSNP. The concentrations of DP92-loaded MSNP and DP92-loaded TPP-MSNP were measured using a UV spectrophotometer (TU-1800, Duksan Tech, Daegu, Korea). To remove unloaded DP92, the sediment was washed 3 times with methanol, and then washed with water 2 times to remove the methanol. 

### 4.7. Characterization of MSNP and TPP-MSNP

#### 4.7.1. N_2_ Sorption Analysis for Surface Area

Surface areas of MSNP or TPP-MSNP were measured by N_2_ adsorption porosimetry using Micromeritics ASAP 2020 (Micromeritics Co., Norcross, GA, USA). For the N_2_ measurements, 30 mg of each sample was degassed under vacuum for 24 h at 40 °C. The Brunauer–Emmett–Teller (BET) method was used to determine the surface area (SBET).

#### 4.7.2. Transmission Electron Microscopy and Fourier Transform Infrared Spectroscopy

A drop of the solution (0.001% (w/w) aqueous solution) was placed on a Formvar-coated copper grid, and the solution was allowed to evaporate under ambient conditions. After 5 min, the excess solution was removed using filter paper. The dried specimens were observed using a TEM JEOL-JEM 2010 instrument (JEOL, Tokyo, Japan).

A Fourier transform infrared spectroscopy (FT-IR) experiment was performed with Nicolet 5700 IR spectrometer (Thermo Electron, Madison, WI, USA). The range was carried out from 500 to 4000 cm^−1^.

#### 4.7.3. Particle Diameter, Polydispersity Index, and Zeta Potential Analysis

The particle diameter, polydispersity index (PI), and zeta potential of MSNP or TPP-MSNP were analyzed using a dynamic light scattering method using Zetasizer ELSZ-2000 (Otsuka Electronics Co., Ltd., Osaka, Japan). All samples were diluted 10-fold with distilled water or F/2 media before measuring particle diameter, polydispersity index, and zeta potential. 

#### 4.7.4. Determination of Encapsulation Efficiency of DP92-Loaded MSNP

The encapsulation efficiency of DP92 was analyzed using UV spectroscopy. The detection wavelength was 237 nm. The supernatant collected during the preparation process was used to analyze the encapsulation efficiency. The encapsulation efficiency was calculated according to Equation (1):

(1) $$\mathrm{EE}\;(\%)=(1-\frac{\mathrm{Amount}\;\mathrm{of}\;\mathrm{DP}92\;\mathrm{in}\;\mathrm{supernatant}\left(\mathrm{mg}\right)}{\mathrm{Amount}\;\mathrm{of}\;\mathrm{initial}\;\mathrm{feeded}\;\mathrm{DP}92\left(\mathrm{mg}\right)})\times 100$$

#### 4.7.5. In Vitro Release of DP92-Loaded MSNP

In vitro release of DP92 from MSNP or TPP-MSNP was carried out in an algal culture medium (containing 1% (*w*/*v*) Tween 80 for sink condition) presented in Section 4.8. “Algal culture conditions”. The 2 mL of MSNP or TPP-MSNP solution with a final DP-92 concentration of 10 mM was added into a dialysis bag (12 kDa cut-off), and the bag was placed and rotated in the algal culture medium at 20 °C under sink condition. At predetermined time intervals, 1 mL of the medium was taken out and replaced with an equal volume of the fresh medium to maintain the sink condition. The concentration of DP92 in the taken samples was analyzed using an HPLC system (Azura, Germany). The HPLC system consisted of pumps (P 6.1L), an autosampler (AS 6.1L), and a UV detector (DAD 2.1L) (Azura, Germany). A C_18_ column (Luna C18, 4.6 mm × 150 mm, 5 μm; Phenomenex, Torrance, CA, USA) was used and heated to 40 °C in a column oven. DP92 was eluted with a mobile phase consisting of 10 mM ammonium acetate/acetonitrile (30:70, *v*/*v* %), and the detection wavelength was fixed at 237 nm. The flow rate was 1.0 mL/min. The injection volume was 10 μL.

### 4.8. Algal Culture Conditions

The F/2 medium was filtered through 0.45 μm and 0.2 μm membrane filters and autoclaved for 15 min at 121 °C. The filtered medium was adjusted to a pH of 8 via shaking at 23 °C and then further incubated under shaking before cultivating the cells. Polystyrene cell culture flasks were obtained from SPL Life Sciences Co., Ltd. (Gyeonggi, Korea). To evaluate the activity of the algicide, two different kinds of algae, *Heterocapsa circularisquama* (HC) and *Heterosigma akashiwo* (HA), were obtained from the Korea Marine Microalgae Culture Center (KMMCC). Harmful algae were grown in a culture flask at 20 °C under a light/dark cycle of 10 h light/14 h dark, a light intensity of 40 PPFD, and a salinity of 33 psu in F/2 media [4].

### 4.9. Fluorescence Microscopy Observation

To observe migration into algae, coumarin-6 labeled MSNP or TPP-MSNP were used. The fluorescence-labeled MSNP or TPP-MSNP were prepared by modifying the method [40]. Briefly, MSNP (5 mg) or TPP-MSNP (5 mg) were mixed with coumarin-6 (5 mg) in methanol (1 mL). The mixtures were stirred for 24 h, followed by centrifugation at 5000 rpm for 5 min. Then, the sediment was washed as described in “Preparation of DP92 loaded MSNP and TPP-MSNP”. *H. circularisquama* or *H. akashiwo* algae (180 μL) were transferred to 96 wells, mixed with coumarin-6 labelled MSNP or TPP-MSNP (20 μL), and incubated under algae culture conditions for 24 h. The incubated algae were observed under a fluorescence microscope (Olympus, Tokyo, Japan).

### 4.10. Fluorescence Absorbance Measurement

To evaluate the transition into algae, coumarin-6 labeled MSNP or TPP-MSNP were used. Coumarin-6 labeled TPP-MSNP or MSNP were prepared using the same method described in “Fluorescence microscopy”. Coumarin-6 labeled TPP-MSNP or MSNP (500 μL) were mixed with algae (4.5 mL) in a conical tube. Coumarin-6-labeled TPP-MSNP or MSNP-treated algae were incubated in an incubator for 24 h. To evaluate the degree of penetration of algae, coumarin-6 inside algae was isolated using the following isolation method. The mixture was centrifuged at 800 rpm for 5 min, and the supernatant was removed to separate the algae from the TPP-MSNP and MSNP labeled with coumarin-6 that did not transit into the algae. After adding the new culture medium, the mixture was sonicated with a probe sonicator (VCX 500, Sonics & Materials, INC., Newtown, CT, USA) for 5 min to rupture the algal cell wall. The sonicated solution was centrifuged at 800 rpm for 5 min. The fluorescence absorbance of the supernatant was measured using a microplate analysis system (Spectramax M3, Molecular Devices, Sunnyvale, CA, USA). 

### 4.11. Algicidal Activity of DP92-Loaded MSNP and TPP-MSNP

To estimate the algicidal activity of DP92-loaded MSNP, TPP-MSNP, and DP92 dissolved in DMSO, *H. akashiwo* and *H. circularisquama* were treated. The final concentrations were adjusted to 1, 0.5, 0.4, 0.2, 0.1, and 0.05 µM in *H. akashiwo* and 2, 1, 0.5, 0.4, 0.2, and 0.1 µM in *H. circularisquama*. To adjust the concentrations, an F/2 medium was used as the dilution solution. At 96 wells, 180 µL of algae were taken, and 20 µL of DP92-loaded MSNP were added to each of the 180 µL of algae. The treated algae were incubated for 24 h and the number of live algae was counted using optical microscope at ×400 magnification (Olympus, Tokyo, Japan). Algicidal activity was calculated according to Equation (2):

(2) $$\mathrm{Algicidal}\;\mathrm{activity}\;(\%)=\left(1-\frac{\mathrm{Tt}}{\mathrm{Ct}}\right)\times 100$$

where T (treatment) and C (control) are the cell densities and t is the inoculation time (day).

### 4.12. DP92 Internalization Efficiency of DP92-Loaded MSNP or TPP-MSNP

To evaluate the DP92 internalization into algae, DP92-loaded MSNP or TPP-MSNP were used. DP92-loaded MSNP or TPP-MSNP were mixed with HABs (*H. akashiwo* or *H. circularisquama*) in a conical tube. The mixtures (10 mL) were adjusted to 1 μM and left for 2 h for the transition of DP92 into algae. Then, DP92 was isolated from the algae using the method described in “Fluorescence absorbance measurement”. The isolated samples were lyophilized for concentration. The lyophilized sample was dissolved in methanol (1 mL). Then, the concentration of DP92 was measured using an HPLC system described in Section 4.7.5. “In vitro release of DP92-loaded MSNP”.

### 4.13. Statistical Analysis

IC_50_ was calculated using nonlinear regression, and the data were fitted to a sigmoidal dose–response relation using the program GraphPad Prism (ver. 5.01; GraphPad Software, Inc., San Diego, CA, USA), according to Equation (3):

(3) Y=100(1+10^(LogIC50−X (×HillSlope)))

where X is the log of dose or concentration and Y is the normalized response, 0 to 100%, increasing as X increases. The log IC_50_ is in the same log units as X, and HillSlope is the slope factor or “hill slope”, which has no unit.

Unpaired data were statistically analyzed using Student’s t-test to compare two mean values; to compare more than two mean values, one-way analysis of variance (ANOVA) followed by Tukey’s multiple-comparison test was used. All data are expressed as mean ± standard deviation. A *p*-value < 0.05 was considered statistically significant in all analyses.

## 5. Conclusions

TPP-MSNP has been developed to entrap DP92 and to increase its algicidal activity. The results suggest that TPP successfully functionalizes the MSNP. TPP-MSNP effectively solubilizes DP92. TPP-MSNP loading DP92 has shown higher algicidal activity than the control and MSNP loading DP92. Through fluorescence microscopy and fluorescence absorption measurements, we have identified that TPP-MSNP successfully targets HABs.

The solubilization of DP92, the induction of the charge interaction between the TPP-MSNP and the HABs’ surface, and the accelerating penetration of DP92 into HABs have demonstrated enhanced algicidal activity of DP92. Therefore, we have successfully developed a DP92 in TPP-MSNP that is easier to prepare, less expensive, and enhances the algicidal activity of DP92.

## Figures and Tables

**Table 1:** Diameter, polydispersity index (PI), and zeta potential of MSNP or TPP-MSNP. Data are presented as mean ± SD (*n* = 3).

	Mean Diameter (nm)	PI	Zeta Potential (mV)
MSNP	126.43 ± 9.46	0.25	−31.67 ± 1.14
MSNP/DP92	132.37 ± 4.85	0.24	−29.20 ± 0.95
TPP-MSNP	104.27 ± 2.05	0.20	25.41 ± 1.53
TPP-MSNP/DP92	110.73 ± 5.66	0.21	26.63 ± 1.07

**Table 2:** Encapsulation efficiency (%EE) of DP92 in MSNP or TPP-MSNP. Data are presented as mean ± SD (*n* = 3).

Feeding Amount of DP92	EE (%)	
	MSNP	TPP-MSNP
10 mg	80.3 ± 4.6	81.9 ± 3.9
15 mg	85.2 ± 4.3	88.7 ± 2.5
20 mg	77.4 ± 6.2	73.8 ± 3.5

**Table 3:** Fluorescence absorbance intensity of coumarin-6 labeled MSNP or TPP-MSNP in HABs. Data are presented as mean ± SD (*n* = 3). *^,#^ *p* < 0.01 compared with MSNP.

	*H. akashiwo*	*H. circularisquama*
MSNP	120.49 ± 3.52	177.69 ± 2.42
TPP-MSNP	171.46 ± 5.19 *	230.64 ± 4.62 ^#^

**Table 4:** IC_50_ values of DP92-loaded MSNP or TPP-MSNP in HABs. Data are presented as mean ± SD (*n* = 3). *^,#^ *p* < 0.01 compared with the control group.

	IC_50_ (μM)	
	*H. akashiwo*	*H. circularisquama*
Control	0.27 ± 0.02	1.90 ± 0.09
MSNP	0.16 ± 0.03 *	0.29 ± 0.02 ^#^
TPP-MSNP	0.04 ± 0.01 *	0.10 ± 0.02 ^#^

**Table 5:** Cellular internalization of DP92-loaded MSNP or TPP-MSNP in HABs. Data are presented as mean ± SD (*n* = 3). * *p* < 0.01 compared with MSNP and ^#^ *p* < 0.05 compared with MSNP.

	Cellular Internalization (%)	
	*H. akashiwo*	*H. circularisquama*
MSNP	53.00 ± 6.01	36.12 ± 4.24
TPP-MSNP	83.28 ± 6.00 *	50.94 ± 6.81 ^#^

## Data Availability

Not applicable.
